# Unilateral Presumed Ocular Histoplasmosis Syndrome–Like Multifocal Chorioretinopathy With Type 2 Choroidal Neovascularization in a Patient Without Recognized Endemic Exposure: A Case Report

**DOI:** 10.1155/crop/7020150

**Published:** 2026-11-14

**Authors:** Jennifer Abou Chaar, Anthony Daher, Yehya Tlaiss, Mahmoud Jaroudi

**Affiliations:** ^1^ Department of Ophthalmology Faculty of Medicine and Medical Sciences University of Balamand Beirut Lebanon balamand.edu.lb; ^2^ Department of Ophthalmology Clemenceau Medical Center Beirut Lebanon cmc.com.lb

**Keywords:** aflibercept, inflammatory choroidal neovascularization, multifocal choroiditis, multimodal imaging, presumed ocular histoplasmosis syndrome, punctate inner choroidopathy

## Abstract

Presumed ocular histoplasmosis syndrome (POHS) is a clinical diagnosis defined by peripapillary atrophy, punched‐out chorioretinal scars, and choroidal neovascularization (CNV) in an eye without intraocular inflammation, and is classically linked to residence in regions endemic for *Histoplasma capsulatum*. A 24‐year‐old healthy, emmetropic woman presented with a 6‐month history of a central scotoma in the left eye. Best corrected visual acuity (BCVA) was 20/20 in the right eye and 20/100 in the left. She had lived only in Lebanon and France and had no recognized exposure to a highly endemic region. The left fundus showed peripapillary atrophy, multiple punched‐out midperipheral chorioretinal scars including a curvilinear (Schlaegel) streak, and a juxtafoveal Type 2 CNV, without anterior chamber or vitreous cells; the right eye was clinically normal. Indocyanine green angiography showed hypocyanescent lesions exceeding those visible clinically, a finding shared with multifocal choroiditis and punctate inner choroidopathy. An infectious and inflammatory work‐up, including *Histoplasma* serology, was negative. Three loading doses of intravitreal aflibercept restored BCVA to 20/20. Asymptomatic recurrence 1 month later (BCVA 20/50) was controlled with a treat‐and‐extend regimen; over 20 months and 16 visits, the eye remained free of intraocular inflammation, and the fellow eye remained clinically normal. The case fulfills Standardization of Uveitis Nomenclature classification criteria for multifocal choroiditis with panuveitis; because its etiology could not be established, we describe its clinical phenotype as POHS‐like. Such a phenotype can occur outside recognized endemic areas; its neovascular complication responds well to anti‐VEGF therapy but may recur, warranting structured imaging surveillance.

## 1. Introduction

Presumed ocular histoplasmosis syndrome (POHS) is a clinical diagnosis characterized by a triad of peripapillary atrophy, discrete punched‐out chorioretinal scars (“histo spots”), and choroidal neovascularization (CNV), occurring in an eye free of anterior chamber and vitreous inflammation [1–5]. It is classically associated with prior exposure to *Histoplasma capsulatum*, a dimorphic fungus most prevalent in the Ohio and Mississippi River valleys of the United States, where histoplasmin skin‐test positivity is common in the adult population [6, 7]. Beyond North America, *Histoplasma* is recognized in parts of Central and South America, Africa, and Asia, including India and the Yangtze River basin of China, and sporadic autochthonous infections have been documented in Europe, most notably in the Po River valley of Italy. Its true distribution is therefore wider and less sharply defined than classic maps suggest [7–10].

The qualifier “presumed” reflects genuine etiologic uncertainty. The organism is only rarely recovered from POHS lesions [11], and the same fundus phenotype has been documented in people with no plausible exposure to *H*. *capsulatum*. A Dutch series identified 81 patients who fulfilled every ophthalmic criterion for POHS in a country where the fungus is not endemic [12], and a subsequent etiologic study found no immunologic evidence of previous *Histoplasma* exposure in such patients [13]. Comparable cases have been reported from Germany, Brazil, India, Turkey, and the Middle East [14–18]. On this basis, nonendemic “POHS” is increasingly regarded as lying within the spectrum of idiopathic multifocal choroiditis (MFC) [19, 20], and the two entities can be difficult to separate even for experienced observers [21].

Two features make the present case atypical. First, the patient had no recognized exposure to a highly endemic region. Second, the involvement was clinically unilateral throughout 20 months of follow‐up, whereas in classic POHS chorioretinal scars are usually bilateral even when CNV is confined to one eye [4, 22].

We describe a young woman with a clinically unilateral POHS‐like multifocal chorioretinopathy complicated by Type 2 CNV and documented with multimodal imaging. Rather than assuming the diagnostic label, we weigh the evidence for POHS against its principal mimics, idiopathic MFC and punctate inner choroidopathy (PIC), and classify the case against the Standardization of Uveitis Nomenclature (SUN) criteria. The report′s value is chiefly educational: Nonendemic POHS‐like cases have been documented before, and this case adds multimodal documentation of the phenotype and of its response to anti‐VEGF therapy. This report was prepared in accordance with the CARE guidelines.

## 2. Case Presentation

### 2.1. Patient Information

A 24‐year‐old previously healthy woman presented in August 2023 with a 6‐month history of central visual disturbance in the left eye (OS), described as a fixed dark spot with progressive central blurring that began while she was living in France. She reported no photopsia, floaters, pain, redness, or systemic symptoms.

She was born and raised in Beirut, Lebanon, in an urban setting, and had lived in Paris, France, for 2 years before symptom onset; she had never lived elsewhere. Her travel had been confined to Lebanon and France, and she had never visited North, Central, or South America, sub‐Saharan Africa, South or Southeast Asia, or China. She denied entering caves or abandoned buildings, exposure to bird or bat guano, poultry farming, soil excavation, demolition, or agricultural work. Because *Histoplasma* has a broad and incompletely mapped distribution, with sporadic autochthonous infections reported in Europe [9, 10], this history establishes the absence of recognized exposure to a highly endemic region rather than proof that exposure never occurred.

She was a lifelong nonsmoker, which is of interest because smoking is the strongest identified risk factor for CNV in endemic POHS [23]. She was emmetropic. There was no history of immunosuppression, HIV risk behavior, autoimmune disease, or prior ocular inflammation, trauma, or surgery.

### 2.2. Ophthalmic Examination

Best corrected visual acuity (BCVA) was 20/20 in the right eye (OD) and 20/100 in the OS, with no improvement on refraction or pinhole. Pupils were equal and reactive with no relative afferent pupillary defect. Intraocular pressure was 12 mm Hg OD and 13 mm Hg OS. Anterior segment examination was normal bilaterally, with clear corneas, deep and quiet anterior chambers, and clear lenses. There were no anterior chamber cells, no anterior vitreous cells, and no vitreous haze in either eye.

#### 2.2.1. OD

The optic disc was normal with sharp margins, the vasculature and macula were unremarkable, and the peripheral retina was attached and free of lesions on dilated indirect ophthalmoscopy (Figure 1A).

**Figure 1:**
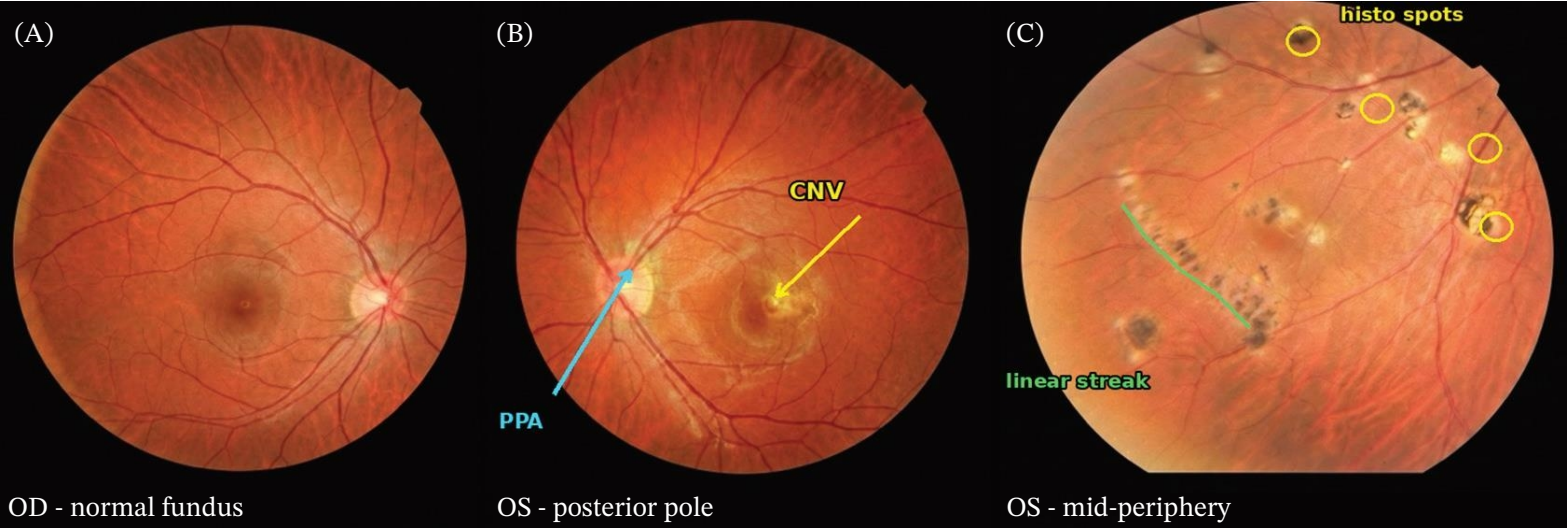
Color fundus photography. (A) Right eye: normal disc, macula, and vasculature. (B) Left eye, posterior pole: peripapillary atrophy (PPA) (blue arrow) and a juxtafoveal grey‐yellow subretinal lesion with surrounding pigmentary change consistent with choroidal neovascularization (CNV) (yellow arrow). (C) Left eye, midperiphery: multiple round punched‐out chorioretinal scars (yellow circles), several arranged in a curvilinear chain forming a linear (Schlaegel) streak (green tracing). No vitreous haze is present in any field.

#### 2.2.2. OS

There was a discrete rim of peripapillary chorioretinal atrophy. Multiple round, well‐circumscribed, and yellow‐white punched‐out chorioretinal scars were present, predominantly in the midperiphery, several with pigmented margins. A number of these lesions were arranged in a curvilinear chain parallel to the ora serrata, forming a linear (Schlaegel) streak, a recognized feature of the POHS phenotype that has also been described in nonendemic Dutch patients [24]. At the posterior pole, there was a grey‐yellow juxtafoveal subretinal lesion with a surrounding halo of subretinal fluid, suspicious for CNV. No vitreous cells, snowballs, vascular sheathing, or active white lesions were seen (Figure 1B,C).

### 2.3. Multimodal Imaging

#### 2.3.1. Optical Coherence Tomography (OCT)

Spectral‐domain OCT of the OS demonstrated a dome‐shaped, moderately reflective subretinal hyperreflective material (SHRM) lesion above an intact but elevated retinal pigment epithelium (RPE), with overlying intraretinal cystoid spaces and adjacent subretinal fluid (Figure 2B). The ellipsoid zone was disrupted over the lesion, and central subfield thickness was increased relative to the fellow eye. Neovascular tissue in the subretinal space above the RPE defines a Type 2 membrane, the pattern characteristic of inflammatory CNV in young patients and distinct from the sub‐RPE (Type 1) growth typical of age‐related macular degeneration [25]. OCT of the OD was normal, with preserved foveal contour and intact outer retinal bands (Figure 2A).

**Figure 2:**
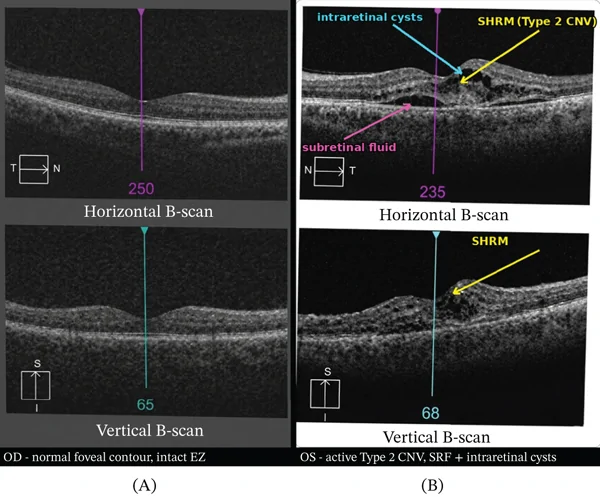
Baseline spectral‐domain optical coherence tomography (horizontal and vertical B‐scans). (A) Right eye: normal foveal contour with an intact ellipsoid zone and no fluid. (B) Left eye: dome‐shaped subretinal hyperreflective material above the retinal pigment epithelium (SHRM) (yellow arrows) with overlying intraretinal cystoid spaces (blue arrow) and adjacent subretinal fluid (magenta arrow), identifying a Type 2 neovascular membrane.

#### 2.3.2. Fluorescein Angiography

Early‐phase fundus fluorescein angiography (FFA) showed a well‐demarcated lacy hyperfluorescent lesion adjacent to the fovea together with multiple punctate hyperfluorescent window defects corresponding to the atrophic scars (Figure 3B,C). Late frames showed progressive leakage from the macular lesion with blurring of its margins and late staining of the chorioretinal scars, without optic disc leakage or retinal vascular staining (Figure 3D). These negative findings argue against active retinal vasculitis or diffuse choroiditis at presentation.

**Figure 3:**
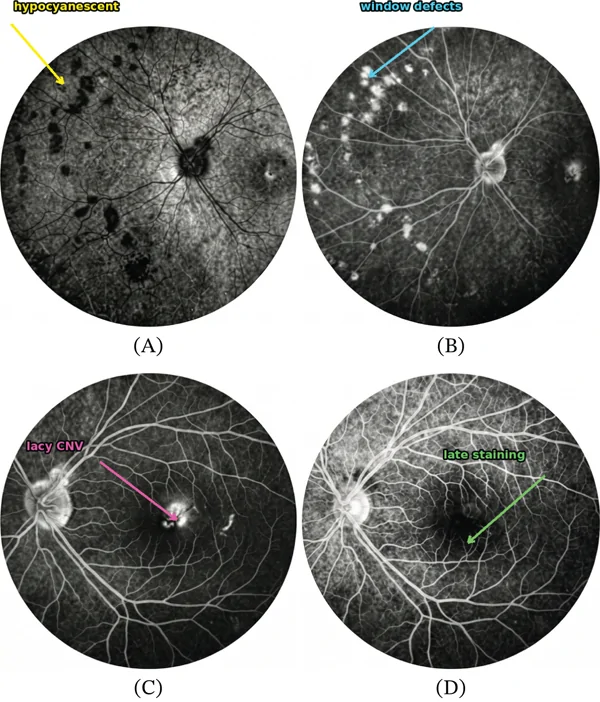
Angiography of the left eye. (A) Indocyanine green angiography: numerous well‐defined hypocyanescent lesions throughout the posterior pole and midperiphery, more numerous than the scars visible on ophthalmoscopy (yellow arrow); this finding is shared with the inflammatory choriocapillaropathies and is not specific for POHS. (B) Early‐phase fluorescein angiography: punctate hyperfluorescent window defects corresponding to the atrophic chorioretinal scars (blue arrow). (C) Early‐phase fluorescein angiography, macula: well‐demarcated lacy hyperfluorescent juxtafoveal lesion consistent with a Type 2 neovascular membrane (magenta arrow). (D) Late phase: leakage with blurring of the lesion margins and late staining of the surrounding scars (green arrow), without optic disc leakage or retinal vascular staining.

#### 2.3.3. Indocyanine Green Angiography (ICGA)

ICGA showed numerous well‐defined hypocyanescent lesions throughout the posterior pole and midperiphery, more numerous than the scars visible on ophthalmoscopy, with focal hypercyanescence at the CNV complex (Figure 3A). Hypocyanescent lesions in excess of clinically visible scars are a recognized feature of the inflammatory choriocapillaropathies, including MFC and PIC, in which they reflect choriocapillaris nonperfusion and typically regress with treatment; atrophic scars, by contrast, remain hypocyanescent and unchanged over time [26, 27]. ICGA was not repeated during follow‐up, so a single baseline study could not determine which process these lesions represented. We therefore regard the ICGA findings as compatible with MFC/PIC and did not use them as evidence in favor of POHS.

#### 2.3.4. Fellow‐Eye Imaging

Baseline color fundus photography and OCT of the OD were normal (Figures 1A and 2A). Fluorescein angiography of the OD, performed in France before referral, was normal; a copy of the angiogram is held in the patient′s record. ICGA, fundus autofluorescence, and widefield imaging of the OD were not obtained. At the final visit, the OD was documented by dilated fundus examination and OCT, both of which were normal.

### 2.4. Investigations

A systemic infectious and inflammatory work‐up was performed to exclude alternative causes of multifocal chorioretinal scarring with CNV. Serology for *Toxoplasma gondii* (IgG and IgM), syphilis (rapid plasma reagin and treponemal testing), HIV, and *Bartonella henselae* was negative. An interferon‐*γ* release assay and chest radiography showed no evidence of latent or active tuberculosis. Serum angiotensin‐converting enzyme, lysozyme, antinuclear antibodies, rheumatoid factor, erythrocyte sedimentation rate, and C‐reactive protein were within normal limits. *Histoplasma* serology (complement fixation and immunodiffusion) was negative. Histoplasmin skin testing was not available locally, and being of limited specificity, was not pursued.

A negative *Histoplasma* serology does not exclude POHS: Serologic and skin‐test positivity reflect remote exposure, both may be absent years after a self‐limited primary infection, and POHS remains a clinical diagnosis [2, 13].

### 2.5. Diagnosis and Differential Diagnosis

A diagnosis of active Type 2 CNV arising in a clinically unilateral POHS‐like multifocal chorioretinopathy of the OS was made.

#### 2.5.1. SUN Classification

The SUN classification criteria for MFCPU require (1) MFC with a predominant lesion size greater than 125 *μ*m; (2) lesions outside the posterior pole, with or without posterior involvement; and either (3) punched‐out atrophic scars or (4) more than minimal anterior chamber and/or vitreous inflammation [28]. The case meets Criteria 1, 2, and 3: On caliper measurement of fundus images, the lesions were predominantly larger than 250 *μ*m (and therefore larger than 125 *μ*m), were located predominantly in the midperiphery, and were punched‐out atrophic scars; Criterion 4 was not met. The case therefore fulfills SUN classification criteria for MFCPU. Syphilis, sarcoidosis, and tuberculosis, the principal infectious and granulomatous alternatives, were excluded by the work‐up described in Section 2.4. Because POHS is not among the diseases for which SUN classification criteria were developed, and classification criteria describe a phenotype without establishing its cause, we report the case as MFCPU by classification with a POHS‐like clinical phenotype. The remaining differential diagnosis is summarized below, and the relationship to idiopathic MFC and PIC is discussed in Section 3.2:

• Idiopathic MFC. As stated above, the case meets SUN classification criteria for MFCPU. Features atypical for idiopathic MFC were the persistent absence of intraocular inflammation and of new lesions over 20 months, emmetropia, peripapillary atrophy, a Schlaegel streak, and clinically unilateral disease (Section 3.2).
• PIC. SUN classification criteria for PIC require punctate choroidal lesions smaller than 250 *μ*m with absent to minimal intraocular inflammation and posterior pole involvement, with or without midperipheral lesions; the disease typically affects young myopic women [29]. These criteria were not met because the lesions were predominantly larger than 250 *μ*m on caliper measurement and located mainly in the midperiphery. These are the same features that satisfy Criteria 1 and 2 for MFCPU.
• Serpiginous and serpiginous‐like (tuberculous) choroiditis. Characterized by geographic, contiguous, centrifugally progressive lesions rather than discrete round scars; tuberculosis was not supported by the interferon‐*γ* release assay and chest radiography.
• Ocular toxoplasmosis. Typically produces a solitary pigmented scar, often with satellite lesions and vitritis on reactivation; the lesions here were multiple and discrete, there was no vitritis, and serology was negative.
• Syphilitic chorioretinitis. Usually accompanied by intraocular inflammation and placoid or multifocal active lesions; treponemal and nontreponemal tests were negative.
• Sarcoidosis. Multifocal choroidal granulomas are usually accompanied by vitritis, periphlebitis, or systemic findings; there were none, and serum angiotensin‐converting enzyme, lysozyme, and chest radiography were normal.
• Birdshot chorioretinopathy. Bilateral ovoid cream‐colored lesions in older adults with vitritis; the unilateral, punched‐out, and pigmented scars in a young patient were inconsistent with this diagnosis.
• Myopic CNV. Excluded by the emmetropic refraction and the absence of posterior staphyloma or lacquer cracks.

### 2.6. Treatment, Follow‐Up, and Outcomes

The patient received three monthly loading doses of intravitreal aflibercept 2 mg in 0.05 mL. One month after the third injection, OCT demonstrated complete resolution of subretinal fluid and intraretinal cysts, flattening of the RPE, and regression of the SHRM into a compact, densely reflective plaque consistent with fibrotic involution of the neovascular complex. Foveal contour was restored, and BCVA improved from 20/100 to 20/20 OS (Figure 4).

**Figure 4:**
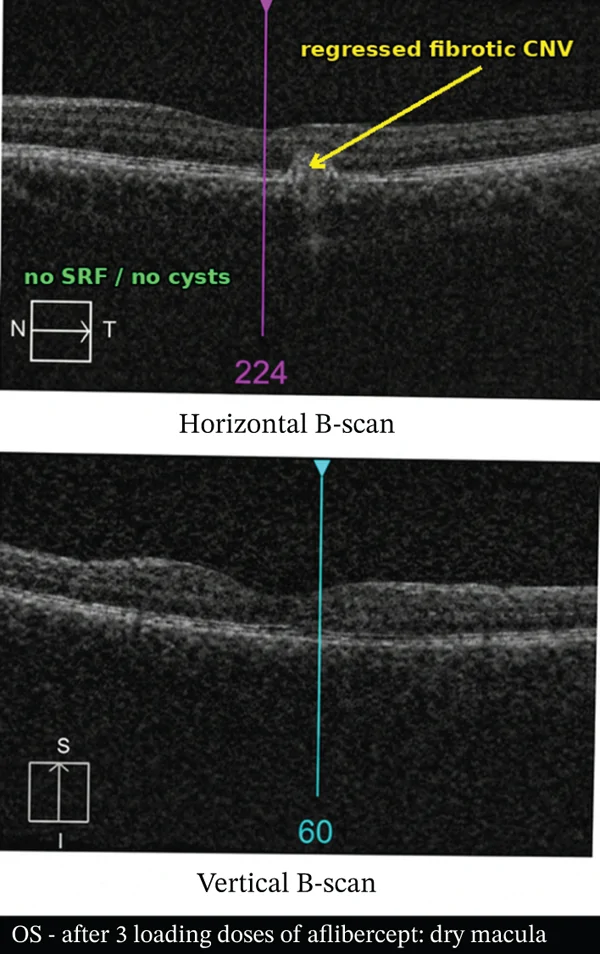
Left eye optical coherence tomography after three loading doses of intravitreal aflibercept. The subretinal hyperreflective material has regressed into a compact, densely reflective subfoveal plaque consistent with fibrotic involution of the neovascular complex (yellow arrow). Subretinal fluid and intraretinal cysts have resolved and the foveal contour is restored; best corrected visual acuity recovered to 20/20.

One month after this dry examination, routine OCT detected recurrent subretinal fluid with a decline in BCVA to 20/50 OS, although the patient was asymptomatic. Aflibercept was resumed and a treat‐and‐extend regimen was instituted, achieving anatomical stability at injection intervals of 6 to 8 weeks.

Over a total follow‐up of 20 months the patient attended 16 visits. No further exudative recurrence occurred once the treat‐and‐extend interval had been established. Serial OCT showed a dry macula between injections, with BCVA ranging from 20/20 to 20/60 OS; the variability was attributed to the subfoveal fibrotic plaque and associated ellipsoid zone disruption. Slit‐lamp biomicroscopy and dilated fundus examination were performed at every visit, and at none of the 16 visits were anterior chamber cells, vitreous cells or haze, vascular sheathing, or new or active chorioretinal lesions observed. The OD remained normal on examination throughout. The patient tolerated all injections without ocular or systemic adverse events, and adherence to the schedule was complete.

## 3. Discussion

### 3.1. A POHS‐Like Phenotype Outside Recognized Endemic Areas

Our patient had the complete ophthalmic phenotype of POHS—peripapillary atrophy, punched‐out midperipheral scars, a linear streak, and CNV in a quiet eye—without recognized exposure to an endemic region and with negative *Histoplasma* serology. Comparable nonendemic cases have been described in Europe, South America, India, Turkey, and the Middle East [12, 14–18]. Two caveats apply to the epidemiology. First, the range of *Histoplasma* is broader than the classic North American map, and autochthonous infections occur sporadically in Europe [8–10], so the absence of recognized exposure is not proof of no exposure. Second, in nonendemic cohorts such as the Dutch series, the lack of immunologic evidence of exposure suggests that a POHS phenotype may arise without *H*. *capsulatum* at all [12, 13].

The disease also remained clinically unilateral. In classic POHS, chorioretinal scars are usually bilateral even when CNV affects one eye [4, 22], so a clinically normal fellow eye over 20 months is unusual. This observation should be interpreted with caution. Fluorescein angiography of the OD performed in France was normal, but ICGA, fundus autofluorescence, and widefield imaging of the OD were not obtained, so subclinical lesions, which could appear as hypocyanescent or hypoautofluorescent spots, cannot be excluded, and unilaterality rests on clinical rather than complete angiographic documentation. A normal‐appearing fellow eye therefore does not exclude a POHS‐like phenotype, nor does it remove the need for fellow‐eye surveillance, because fellow eyes in POHS carry a measurable risk of subsequent neovascularization [22].

The peripheral linear (Schlaegel) streak, although a classic feature of POHS described in large series, has received relatively little emphasis in nonendemic reports [24]. Such lesions are easily missed on posterior‐pole photography, underscoring the value of widefield imaging or scleral depression.

### 3.2. POHS‐Like Phenotype Versus Idiopathic MFC and PIC

The central question is how this case relates to POHS and to idiopathic MFC and PIC. The conditions share punched‐out chorioretinal scars, peripapillary changes, and CNV; MFC was originally described as a syndrome that mimics ocular histoplasmosis [30], and in a masked study, experienced observers could not reliably separate inactive cases of POHS and MFC on fundus photographs and fluorescein angiograms [21]. As detailed in Section 2.5, the case fulfills SUN classification criteria for MFCPU: MFC with a predominant lesion size greater than 125 *μ*m, lesions outside the posterior pole, and punched‐out atrophic scars [28]. It does not fulfill criteria for PIC, which require punctate lesions smaller than 250 *μ*m with posterior pole involvement [29]. The lesion size and midperipheral distribution that exclude PIC are the same features that satisfy the MFCPU criteria.

Classification as MFCPU describes the phenotype but does not establish its etiology, and several features were atypical for idiopathic MFC and recall the POHS phenotype. The eye remained free of anterior chamber and vitreous inflammation at all 16 visits over 20 months, and no new or active chorioretinal lesions developed, whereas MFC commonly follows a relapsing course with recurrent inflammation and new lesions [19, 30]. The scars were accompanied by peripapillary atrophy and a Schlaegel streak, features classically associated with POHS [3, 24]. Finally, the neovascular membrane was controlled with anti‐VEGF monotherapy, without corticosteroids or immunosuppression.

Against these features, baseline ICGA showed hypocyanescent lesions in excess of the clinically visible scars. In MFC and PIC, such lesions reflect choriocapillaris nonperfusion and typically regress with anti‐inflammatory treatment, whereas atrophic scars remain hypocyanescent and unchanged [26, 27]. Without a repeat study, we cannot determine which applies here, and the ICGA appearance is therefore compatible with MFC/PIC. Negative *Histoplasma* serology does not resolve the question in either direction, because serologic and skin‐test responses reflect remote exposure and may wane over time [2, 13].

Taken together, the case fulfills SUN classification criteria for MFCPU and has a clinical phenotype resembling POHS. The absence of recognized exposure and the negative serology preclude attributing it to *H*. *capsulatum*, and the overlapping ICGA findings are consistent with the view that nonendemic POHS‐like disease lies within the MFC spectrum [19, 20]. We therefore describe it as a POHS‐like multifocal chorioretinopathy that fulfills MFCPU classification criteria. For the individual patient, the label matters less than the management decision it drives, which in this case was determined by the persistent absence of intraocular inflammation.

### 3.3. Management and Surveillance

Because there was no intraocular inflammation, anti‐VEGF monotherapy was considered appropriate; in the inflammatory choroiditides, corticosteroid or immunomodulatory therapy may be required in addition to treatment of the neovascular complex. Continued examination for intraocular inflammation remains important in such patients, as its emergence would favor MFC and prompt anti‐inflammatory treatment. The response was typical of inflammatory and POHS‐associated CNV, which often responds more briskly and completely to vascular endothelial growth factor (VEGF) blockade than neovascular age‐related macular degeneration, frequently with recovery of visual acuity [31–34]. Aflibercept was selected on the basis of availability; published experience in this setting includes bevacizumab, ranibizumab, and aflibercept, with no demonstrated superiority of any one agent [31–34].

Recurrence, however, is common. In our patient, exudation returned within a month of a completely dry macula even though she was asymptomatic; had follow‐up been symptom‐driven, the recurrence would probably have been detected later and the fibrotic plaque might have been larger. A treat‐and‐extend regimen, consistent with long‐term data in inflammatory ocular neovascularization [35], achieved durable control at 6‐ to 8‐week intervals. Young patients with this condition may face many years of surveillance, and expectations should be set accordingly at the outset.

### 3.4. Limitations

This is a single case with 20 months of follow‐up. Histoplasmin skin testing was unavailable, and no tissue diagnosis was obtained, so the etiology remains, as the name concedes, presumed. ICGA was not repeated after treatment, which would have helped determine whether the hypocyanescent lesions represented active choriocapillaris involvement or established atrophy. Fundus autofluorescence was not performed and would have better delineated RPE involvement, and OCT angiography, which can demonstrate the neovascular network noninvasively, was not available. Fellow‐eye documentation was limited: The OD was imaged by color fundus photography, OCT, and fluorescein angiography, but ICGA, fundus autofluorescence, and widefield imaging were not obtained, so subclinical involvement cannot be excluded and unilaterality is a clinical observation. The fellow eye may also develop lesions with longer follow‐up, and the unilateral course reported here applies to the 20‐month observation period.

## 4. Conclusion

A clinically unilateral POHS‐like multifocal chorioretinopathy complicated by Type 2 CNV, fulfilling SUN classification criteria for MFCPU, can occur in a patient without recognized exposure to a highly endemic region. In this case, the diagnosis was clinical and remains etiologically unproven: The phenotype comprised peripapillary atrophy, punched‐out midperipheral scars, a Schlaegel streak, a persistently quiet eye, and Type 2 CNV, whereas *Histoplasma* serology was negative, and ICGA findings overlapped with those of MFC and PIC. Clinicians outside endemic zones should recognize this phenotype rather than discount it on geographic grounds, while recognizing that such cases meet classification criteria for MFCPU and may lie on the same spectrum as idiopathic MFC. Multimodal imaging of both eyes and longitudinal examination for inflammation are essential both for this distinction and for detecting asymptomatic reactivation. The neovascular complication responded well to intravitreal anti‐VEGF therapy but recurred early, supporting structured long‐term OCT surveillance.

**Nomenclature**
BCVA: best corrected visual acuity
CNV: choroidal neovascularization
FFA: fundus fluorescein angiography
ICGA: indocyanine green angiography
MFC: multifocal choroiditis
MFCPU: multifocal choroiditis with panuveitis
OCT: optical coherence tomography
OD: right eye
OS: left eye
PIC: punctate inner choroidopathy
POHS: presumed ocular histoplasmosis syndrome
RPE: retinal pigment epithelium
SHRM: subretinal hyperreflective material
SUN: Standardization of Uveitis Nomenclature
VEGF: vascular endothelial growth factor

## Author Contributions

Jennifer Abou Chaar: conceptualization, investigation, and writing—original draft. Anthony Daher: investigation, data curation, visualization, and writing—review and editing. Yehya Tlaiss: conceptualization, methodology, writing—original draft, writing—review and editing, project administration, and correspondence. Mahmoud Jaroudi: clinical management, resources, validation, supervision, and writing—review and editing.

## Funding

No funding was received for this manuscript.

## Disclosure

All authors read and approved the final manuscript and agree to be accountable for all aspects of the work.

## Ethics Statement

This report describes the routine clinical care of a single patient and did not constitute research on human participants; formal institutional review board approval was therefore not required at our institution. All procedures were performed in accordance with the 1964 Declaration of Helsinki and its later amendments.

## Consent

Written informed consent for publication of clinical details and accompanying images was obtained from the patient. The patient is not identifiable from the text or figures. A copy of the signed consent form is held by the corresponding author and is available to the editor on request.

## Conflicts of Interest

The authors declare no conflicts of interest.

## Supporting information


**Supporting Information** Additional supporting information can be found online in the Supporting Information section.

## Data Availability

The data that support the findings of this study are available from the corresponding author upon reasonable request.
