# Supplementary material for: Unilateral Presumed Ocular Histoplasmosis Syndrome–Like Multifocal Chorioretinopathy With Type 2 Choroidal Neovascularization in a Patient Without Recognized Endemic Exposure: A Case Report
Source: Case Rep Ophthalmol Med. 2026 Nov 14;2026:7020150. doi: 10.1155/crop/7020150 (PMC13768931; doi:10.1155/crop/7020150)
Supplement: Supplementary file 1 [file CROP-2026-7020150-s001.pdf]

CARE Checklist of information to include when writing a case report

CARE case report guidelines

(cc) BY-NC-ND

Topic	Item	Checklist item description	Reported on Line
Title	1	The diagnosis or intervention of primary focus followed by the words "case report"	2-4
Key Words	2	2 to 5 key words that identify diagnoses or interventions in this case report, including "case report"	41-43
Abstract (no references)	3a	Introduction: What is unique about this case and what does it add to the scientific literature?	17-21
	3b	Main symptoms and/or important clinical findings	21-28
	3c	The main diagnoses, therapeutic interventions, and outcomes	28-37
	3d	Conclusion—What is the main "take-away" lesson(s) from this case?	37-40
Introduction	4	One or two paragraphs summarizing why this case is unique (**may include references**)	44-77
Patient Information	5a	De-identified patient specific information.	80-83
	5b	Primary concerns and symptoms of the patient.	80-83
	5c	Medical, family, and psycho-social history including relevant genetic information	84-91
	5d	Relevant past interventions with outcomes	89-91 (none)
Clinical Findings	6	Describe significant physical examination (PE) and important clinical findings.	93-108, 110-115
Timeline	7	Historical and current information from this episode of care organized as a timeline	80, 191-212
Diagnostic Assessment	8a	Diagnostic testing (such as PE, laboratory testing, imaging, surveys).	116-152, 154-162
	8b	Diagnostic challenges (such as access to testing, financial, or cultural)	161-162, 282-287
	8c	Diagnosis (including other diagnoses considered)	168-189
	8d	Prognosis (such as staging in oncology) where applicable	273-280
Therapeutic Intervention	9a	Types of therapeutic intervention (such as pharmacologic, surgical, preventive, self-care)	191
	9b	Administration of therapeutic intervention (such as dosage, strength, duration)	191, 205-207
	9c	Changes in therapeutic intervention (with rationale)	202-207
Follow-up and Outcomes	10a	Clinician and patient-assessed outcomes (if available)	192-196, 202-210
	10b	Important follow-up diagnostic and other test results	192-201, 207-210
	10c	Intervention adherence and tolerability (How was this assessed?)	211-212
	10d	Adverse and unanticipated events	211-212
Discussion	11a	A scientific discussion of the strengths AND limitations associated with this case report	273-280, 282-290
	11b	Discussion of the relevant medical literature **with references**.	216-272
	11c	The scientific rationale for any conclusions (including assessment of possible causes)	221-260
	11d	The primary "take-away" lessons of this case report (without references) in a one paragraph conclusion	292-301
Patient Perspective	12	The patient should share their perspective in one to two paragraphs on the treatment(s) they received	Not obtained
Informed Consent	13	Did the patient give informed consent? Please provide if requested	Yes ☒ No ☐

Manuscript: Atypical Unilateral Presumed Ocular Histoplasmosis Syndrome With CNV in a Non-Endemic Region | Corresponding author: Yehya Tlaiss, MD
